# Ketogenic Diet for Intensive Care Patients: A Scoping Review

**DOI:** 10.3390/nu18121943

**Published:** 2026-06-16

**Authors:** Julia Bryła, Mateusz Szczupak, Sabina Krupa-Nurcek

**Affiliations:** 1 Institute of Nursing, Faculty of Health Sciences and Psychology, Collegium Medicum, University of Rzeszów, 35-310 Rzeszów, Poland; jb130749@stud.ur.edu.pl; 2 Department of Anaesthesiology and Intensive Therapy in the Nicolaus Copernicus Hospital in Gdańsk, 80-803 Gdańsk, Poland; szczupak.mateusz@icloud.com; 3 Department of Surgery, Faculty of Medicine, Collegium Medicum, University of Rzeszów, 35-310 Rzeszów, Poland

**Keywords:** KD, diet, intensive care, intensive therapy

## Abstract

Background: Critical illness leads to profound metabolic, neuroendocrine and immune disorders that affect the prognosis of patients treated in intensive care units (ICUs). The ketogenic diet, a high-fat and low-carbohydrate eating model, is gaining increasing importance as a potential metabolic intervention in the ICU. Preliminary data suggest that the ketogenic diet (KD) may support the control of seizures in a super-refractive epileptic state (SRSE), stabilize glycemia, reduce insulin demand, and modulate the immune response in sepsis. The aim of this review was to present a synthetic presentation of the current state of knowledge regarding use of the KD in intensive care patients. Methods: The review was carried out in accordance with the guidelines of the Joanna Briggs Institute and PRISMA-ScR. PubMed, Scopus, EBSCO, Web of Science, Google Scholar and Cochrane Library databases were searched (10–19 April 2026) using the Population–Concept–Context model. Full-text observational studies, randomized trials and reviews of the use of KDs in ICU patients were included. Data extraction was performed independently by two reviewers. Results: Of the 42 publications identified, seven studies were included in the analysis. The KD was feasible and safe in both critically ill adults and children. In SRSE, most patients achieved stable ketosis within a few days, which often allowed for reduction or discontinuation of anesthetics. In sepsis, the KD led to glycemic stabilization, reduced insulin demand and reduced immune deregulation; in one study, “after day 4, none of the patients in the KD group required insulin treatment.” The KD also showed beneficial effects on cellular bioenergetics and mitochondrial function. The safety profile was acceptable and adverse reactions were manageable with appropriate monitoring. Conclusions: The KD represents a promising, non-pharmacological metabolic intervention in intensive care, particularly in the treatment of SRSE and in the stabilization of glucose metabolism in sepsis and other critical conditions. Despite the growing number of positive clinical observations, the available evidence remains limited due to small samples, heterogeneous protocols, and a lack of randomized trials. Further, well-designed prospective studies are needed to determine optimal KD implementation protocols and identify the patient populations that benefit most.

## 1. Introduction

Critical illness leads to profound metabolic, neuroendocrine and immune disorders that significantly affect the prognosis of patients hospitalized in intensive care units (ICUs). The ketogenic diet, defined as a high-fat, very-low-carbohydrate nutritional regimen that induces a metabolic state of ketosis, provides an alternative energy source in the form of ketone bodies [1]. In recent years, there has been a growing interest in nutritional interventions that can modulate cellular metabolism, improve tissue bioenergetics and support regenerative processes in conditions of severe organ failure. Although interest in metabolic interventions in the ICU is increasing, the current evidence base remains limited, particularly regarding randomized controlled trials. As highlighted in our review, most available studies are observational, retrospective, or based on clinical protocols, and only one randomized controlled trial has evaluated the KD in critically ill patients. In the RCT by Rahmel et al., stable ketosis was achieved in all patients with sepsis, and “after day 4, none of the patients in the KD group required insulin treatment,” while no major metabolic complications were observed. This underscores both the potential of the KD and the need for further high-quality trials [1]. One such strategy is the KD—a high-fat, low-carbohydrate eating model that induces a state of ketosis and provides an alternative source of energy in the form of ketone bodies. Initially used mainly in the treatment of drug-resistant epilepsy, the KD has gained new importance in the context of intensive care, where glucose metabolism disorders, insulin resistance, stress hyperglycemia and bioenergetic mitochondrial failure are key elements of the pathophysiology of critical disease. The KD has been extensively studied in the management of drug-resistant epilepsy, with numerous clinical trials and systematic reviews confirming its efficacy in both children and adults. Classic studies demonstrated significant seizure reduction in pediatric patients, while more recent analyses have reinforced its role as an established therapeutic option in refractory epilepsy [1,2]. These findings form the historical foundation for exploring the KD as a metabolic intervention in other critical neurological conditions, including RSE and SRSE. Pilot studies have shown that ketones can be an effective and safe source of energy in patients with multi-organ failure, supporting tissue function in conditions of impaired use of glucose and fatty acids [1,2]. Beyond their antioxidant properties, ketone bodies exert multiple biological effects relevant to critical illness, including modulation of neurotransmitter balance, stabilization of neuronal membranes, reduction in pro-inflammatory cytokine production, and improvement of mitochondrial efficiency. These mechanisms have been widely described in the context of KD therapy and provide a broader physiological rationale for its potential application in intensive care settings. In the pilot study, McNelly et al. showed that ketogenic nutrition in mechanically ventilated patients is feasible, well-tolerated, and leads to the effective achievement of ketosis, while reducing insulin demand and reducing hypoglycemic episodes. These results suggest that the KD may represent a potential metabolic intervention aimed at improving cellular bioenergetics in the course of critical illness [3].

In ICU settings, the KD is most commonly administered via enteral feeding, using either commercially available ketogenic formulas or individualized mixtures prepared by the hospital pharmacy or clinical nutrition department. The choice of formulation depends on local availability, patient tolerance, and the required macronutrient ratio. Studies included in this review reported the use of both standardized enteral ketogenic products and customized preparations, confirming that the KD can be feasibly implemented in critically ill patients under appropriate metabolic monitoring [2,3]. The second area intensively studied in the context of KD in the ICU is the treatment of refractive and super-refractive epileptic states (RSE/SRSE). Refractory status epilepticus (RSE) is defined as the persistence of seizure activity despite the use of an adequate dose of benzodiazepine and at least one second-line antiepileptic drug, regardless of the duration of the episode. Super-refractory status epilepticus (SRSE), on the other hand, is an epileptic state that persists for at least 24 h after the start of anesthesia therapy or recurs during reduction in or after discontinuation of anesthesia therapy. It is estimated that RSE develops in 23–48% of patients with epilepsy, while SRSE affects about 22% of patients in this group. RSE is more commonly seen in people with new-onset seizures than in patients with previously diagnosed chronic epilepsy. Etiological conditions show significant geographical diversity: in developing countries, infections of the central nervous system and craniocerebral injuries predominate, while in highly developed countries, stroke and sudden discontinuation of antiepileptic drugs are more common causes [3,4]. In situations where standard pharmacological and anesthetic treatment fails, the KD becomes an alternative therapeutic strategy. In a prospective study, Ren et al. reported that the KD has been shown to be effective in 75% of SRSE patients, leading to resolution of seizures within a few days of implementation, with an acceptable safety profile and the possibility of correcting adverse reactions under close clinical supervision [4]. Similar conclusions are presented in clinical guidelines and procedures, which emphasize that the KD can be implemented in ICU settings as an adjunctive therapy, especially in cases resistant to pharmacological treatment [5].

The mechanisms of action of the KD in intensive care are multidimensional. Ketone bodies such as β-hydroxybutyrate exhibit neuroprotective, anti-inflammatory, and antioxidant properties, as well as modulating neurotransmitter expression and stabilizing cell membranes. In critical illness, in which mitochondrial dysfunction, oxidative stress and energy imbalances occur, ketones may be a more effective metabolic fuel than glucose [4,5]. In addition, the KD affects the reduction in pro-inflammatory cytokines, which may be important in the context of systemic inflammatory reaction syndrome and complications such as septic encephalopathy or PICS (Post-Intensive Care Syndrome). However, the introduction of KD in intensive care conditions is associated with numerous practical challenges. These include the need for close monitoring of metabolic parameters, the risk of electrolyte disorders, the possibility of food intolerance and difficulties in maintaining an adequate ratio of macronutrients in enteral or parenteral nutrition [6]. Nevertheless, a growing number of studies indicate that, with proper clinical supervision, the KD can be safely used even in patients in extremely severe conditions.

In light of the above data, the KD appears to be a promising metabolic strategy in intensive care, both in the context of the treatment of neurological disorders and potential bioenergetic support in multi-organ failure. However, further, well-designed clinical trials are needed to determine optimal deployment protocols, long-term safety, and patient groups that can benefit most from this intervention [4,5,6,7].

## 2. Materials and Methods

### 2.1. Study Design

This study was designed as a scoping review with the aim of mapping and summarizing the existing research on the use of the KD in intensive care settings. A scoping review methodology was selected because the available literature is heterogeneous, limited in volume, and has not been previously synthesized. The objective of this review was to identify the types of studies, populations, and interventions described in the literature, rather than to evaluate the effectiveness of the KD [8].

This review has been prepared in accordance with the methodology developed by the Joanna Briggs Institute and based on the guidelines for reporting systematic reviews and meta-analyses in the version dedicated to scoping reviews (PRISMAScR) [9,10]. PRISMA Checklist is avaliable in the Supplementary Materials.

### 2.2. Inclusion and Exclusion Criteria

We formulated a research question that precisely defined the population, the main conceptual assumptions and the context of the analyzed issue. This allowed for a reliable capture of the current state of knowledge regarding the use of the KD in patients treated in intensive care settings.

The inclusion criteria included: all published scientific papers; original articles (both observational and randomized studies), meta-analyses, systematic and narrative reviews; publications available in the full version; research conducted on humans; only works on patients hospitalized in the ICU who were on a KD; articles that described standard operating procedures; and articles published in English.

The exclusion criteria included: case reports, comments, letters to the editor, and book chapters; works without access to the full text; animal studies; publications in languages other than English; and reports of the use of the KD in non-intensive care units.

#### 2.2.1. Population

This review included studies describing the use of a KD in ICU patients. In this review, ICU was defined as a specialized, self-contained department within a hospital designed for patients with life-threatening illnesses or injuries requiring constant, 24/7 monitoring, advanced support, and specialized nursing care. KD is defined as a high-fat, moderate-protein, and very-low-carbohydrate eating plan designed to force the body into a metabolic state called ketosis [3,4,6,7].

#### 2.2.2. Concept

The aim of this study was to present the current state of knowledge regarding the use of the KD in intensive care patients. The focus was on providing a comprehensive overview of the current state of knowledge regarding the benefits and negative effects of the KD in intensive care patients.

#### 2.2.3. Context

The studies included in the review included intensive care unit patients who were treated with a KD.

#### 2.2.4. Types of Studies

The review included a retrospective observational study and reviews of any type of design and methodology used. Only one randomized controlled trial meeting the inclusion criteria was identified, while the remaining studies were observational or descriptive. This distribution reflects the current state of research on KDs in intensive care, where randomized evidence is still scarce.

### 2.3. Search Strategy

The authors conducted a search in the following databases: PubMed, Scopus, EBSCO, Web of Science, Google Scholar and Cochrane Library. The following keywords were used: “intensive care diets”, “KD in ICU”, “benefits of ICU KD”, “benefits of ICU patients”, as well as their various combinations using AND and OR operators. All the discovered publications were pre-selected on the basis of titles and abstracts, which made it possible to reject works unrelated to the subject of the review. The search process started on 10 April 2026 and was completed on 19 April 2026. Summary search of the review is presented in the Table 1. 

### 2.4. Extraction of Data

To compile the data—including key information from the analyzed publications—a form prepared in accordance with the JBI Scoping Review Guidelines [9] was used. The data extraction process, referred to in the scope reviews as “charting the data” [10,11], was carried out independently by two reviewers. A Population–Concept–Context (PCC) scheme was used to identify the appropriate studies. From each article, information such as the name of the first author, year of publication, country, results and key conclusions were obtained. The entire data collation process was done on Microsoft Excel.

### 2.5. Critical Appraisal Process

A scoping review may include an analysis of the available data without the need for a detailed methodological evaluation of the studies included in the study [9].

### 2.6. Process for Including Publications to the Review

**Figure 1:**
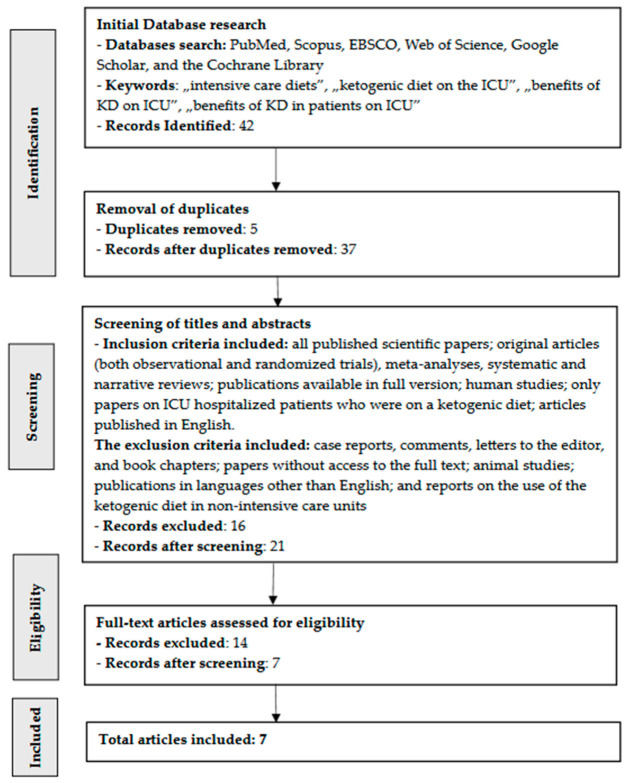
Literature search and selection flowchart for this review.

Our scoping review initially identified a total of 42 articles, of which 7 were ultimately included in the analysis. All articles were closely related to the topic of the scoping review (Figure 1). After removing duplicates (*n* = 5), 37 articles remained. After reviewing the articles according to the inclusion and exclusion criteria (*n* = 16), 21 articles remained. 14 articles did not contain full text and were excluded. As a result, after meeting all requirements, 7 articles were included in the review. The studies were conducted in the USA (*n* = 2), Germany (*n* = 3), China (*n* = 1), and Korea (*n* = 1). The results are presented in Table 2 and Table 3.

## 3. Key Points on the Use of the KD in the Intensive Care Unit

The seven studies included in this scoping review provide the currently available clinical evidence on the use of ketogenic diet therapy in intensive care settings. The following subsections synthesize their main characteristics and findings, focusing on patient populations, indications, dietary protocols, feasibility and safety considerations, metabolic monitoring, and reported clinical outcomes. Given the exploratory nature of the available literature and the methodological scope of this review, these findings are presented as a structured overview of existing evidence rather than as a determination of clinical effectiveness.

### 3.1. KD May Effectively Support the Treatment of SRSE

Super-refractive epileptic state (SRSE) remains one of the most difficult challenges in modern neurological intensive care. Despite the use of multi-drug antiepileptic regimens and continuous infusions of anesthetics, some patients fail to achieve seizure control, which leads to increasing organ failure, metabolic complications and high mortality [14]. In this context, the KD is becoming an increasingly considered metabolic rescue therapy, whose mechanisms of action can support the suppression of seizure activity where pharmacological treatment fails. The KD induces a state of ketosis, in which ketone bodies become the dominant source of energy [15]. A change in the energy substrate of the brain leads to a number of neuroprotective effects: stabilization of neuronal membranes, modulation of ion channels, increase in GABAergic activity and reduction in glutamatergic excitation [16]. In addition, the KD reduces oxidative stress, improves mitochondrial functions and reduces inflammatory processes that often accompany SRSE and can maintain seizure activity [17]. These multidirectional mechanisms make KD work differently from classic antiepileptic drugs, making it a valuable complement to therapy. Clinical data, although still limited, consistently indicate that KD is feasible, safe, and potentially effective in patients with SRSE [16].

In observational studies in adults and children, it has been shown that most patients achieve stable ketosis within a few days, and in a significant proportion SRSE disappears or anesthetics are allowed to discontinue. Importantly, therapeutic effects appear even in patients in whom previous intensive pharmacological treatment has not brought improvement [14,15]. The safety profile of the KD in ICU is acceptable, with mild, manageable metabolic disorders such as hypoglycemia, hyperlipidemia, and acidosis being monitored for the most common and manageable monitoring. A growing body of data also suggests that earlier implementation of the KD may be associated with a higher likelihood of SRSE discontinuation, highlighting the need to develop clear management protocols and increase clinician awareness [16]. The inclusion of the KD as part of the integrated treatment of SRSE can improve prognosis, reduce the duration of anesthetic use, and reduce complications resulting from long-term sedation. The KD represents a promising, non-pharmacological intervention to support the treatment of super-refractive epileptic state. Its unique mechanisms of action, increasing number of positive clinical observations and acceptable safety profile indicate that the KD may play an important role in the therapy of SRSE [14,15,16]. However, further, well-designed clinical trials are needed to pinpoint optimal protocols, implementation time, and patient groups that benefit most from this intervention.

It should be emphasized that six of the seven included studies focused exclusively on refractory or super-refractory status epilepticus. Therefore, the observations presented in this subsection reflect only this subset of the available evidence.

### 3.2. KD May Support Metabolic Control in Patients with Sepsis

Sepsis is a condition characterized by profound metabolic deregulation, including insulin resistance, stress hyperglycemia, lipid metabolism disorders and mitochondrial dysfunction. Standard nutrition based on a high supply of carbohydrates can further exacerbate these disorders, leading to increased insulin demand, glycemic fluctuations and intensification of pro-inflammatory processes [18]. In this context, the KD represents an alternative nutritional strategy that can support metabolic control in patients with sepsis by modulating energy and immunometabolic pathways. KD induces a state of ketosis, in which ketone bodies, especially β-hydroxybutyrate, become the main source of energy [19]. A change in the energy substrate leads to a reduction in glycolysis and a decrease in insulin demand, which can stabilize glycemia and reduce the risk of stress hyperglycemia [20].

In clinical trials, patients with sepsis nourished with a KD have been shown to achieve stable ketosis within a few days, and insulin requirements are significantly reduced, in some cases up to complete discontinuation of insulin after 3–4 days. All findings related to sepsis originate from a single randomized controlled trial by Rahmel et al., which reported that ‘after day 4, none of the patients in the KD group required insulin treatment.’ As this is the only study addressing sepsis in the ICU setting, broader interpretations of metabolic or immunological effects should be considered preliminary. This is particularly important because glycemic fluctuations and high insulin doses are independent factors worsening the prognosis in sepsis [21]. Ketone bodies also exhibit anti-inflammatory and immunomodulatory effects. β-hydroxybutyrate inhibits NLRP3 inflammasome activation, reduces the production of pro-inflammatory cytokines, and promotes a more balanced immune response [22]. In studies in patients with sepsis, the use of a KD was associated with a decrease in gene expression associated with T cell activation and a reduction in the secretion of pro-inflammatory cytokines. This may limit the excessive inflammatory response, which is a key component of the pathophysiology of sepsis and contributes to organ damage. The mechanistic descriptions presented below do not originate from the seven included studies but represent broader theoretical and experimental concepts reported in the literature. These mechanisms provide contextual background but were not directly evaluated in the studies included in this scoping review.

KD also improves mitochondrial functions by increasing the efficiency of oxidative phosphorylation and reducing the production of reactive oxygen species [23]. In sepsis, where mitochondrial dysfunction is one of the main mechanisms leading to organ failure, such an effect may be of significant clinical significance. Ketone bodies are a more efficient fuel than glucose, generating more ATP with a lower oxidative load, which can support cellular regeneration in critical illness conditions [24]. Available data also indicate that KD is feasible and safe in patients with sepsis, provided that metabolic parameters are adequately monitored [23]. No significant adverse reactions such as ketoacidosis or severe lipid disorders were observed, and the safety profile was comparable to standard nutrition. The KD may support metabolic control in patients with sepsis by stabilizing glycemia, reducing insulin demand, improving mitochondrial function, and modulating the immune response [25]. While the results of the studies are promising, further, larger clinical trials are needed to unambiguously determine the impact of the KD on sepsis and organ failure and to establish optimal protocols for its use in intensive care.

### 3.3. KD Can Reduce Insulin Demand and Stabilize Glycemia in Patients in the Intensive Care Unit

Glycemic control is one of the key elements of treatment management in patients in the intensive care unit (ICU). Stress hyperglycemia, insulin resistance and glycemic fluctuations are common in the course of severe diseases such as sepsis, multi-organ failure or severe injuries [26]. Standard nutrition based on a high carbohydrate supply often exacerbates these disorders, leading to increased insulin demand and the risk of hypo- or hyperglycemic episodes. In this context, the KD represents a promising dietary alternative that can support glycemic stabilization and reduction in insulin demand in patients in the ICU [27]. KD induces a state of ketosis, in which ketone bodies become the main source of energy, not glucose. Limiting carbohydrate intake leads to a decrease in glycolysis and a decrease in blood glucose, which directly reduces the need for exogenous insulin [28].

In clinical trials conducted in the ICU, it has been shown that patients fed with a KD achieve stable ketosis within a few days, and insulin demand is significantly reduced—in some cases up to complete insulin discontinuation after 3–4 days of therapy [29]. This is particularly important because intensive insulin therapy is associated with the risk of hypoglycemia, and large fluctuations in glycemia are an independent factor worsening the prognosis. Ketone bodies, especially β-hydroxybutyrate, exhibit additional properties that stabilize glucose metabolism [26,29]. They increase the sensitivity of tissues to insulin, reduce liver glucose production and reduce lipolysis, which is excessively intense under conditions of metabolic stress. As a result, a KD can lead to a more predictable glycemic profile, which facilitates therapy and reduces the risk of episodes of extreme glucose values [30]. KD also affects the reduction in inflammation and oxidative stress, which are key mechanisms driving insulin resistance in the ICU [31,32,33]. β-hydroxybutyrate inhibits the activation of the NLRP3 inflammasome and reduces the secretion of pro-inflammatory cytokines, which may support the improvement of insulin sensitivity and stabilization of metabolism.

The studies also observed a beneficial effect of the KD on mitochondrial function, which further improves the efficiency of the use of energy substrates. Importantly, the available data indicate that the KD is feasible and safe in intensive care, provided that metabolic parameters are properly monitored [32,33]. No serious side effects such as ketoacidosis or serious lipid disorders were observed, and the safety profile was comparable to standard nutrition. The KD may be a valuable tool to support glycemic control in patients in the ICU [27,29]. By reducing insulin demand, stabilizing glycemia, improving insulin sensitivity and modulating the inflammatory response, the KD can contribute to improving treatment outcomes. While the results of the studies are promising, further, well-designed clinical trials are needed to unambiguously determine the optimal protocols for use of the KD in intensive care [32,34].

### 3.4. KD May Support Discontinuation of Anesthetics and Shorten the Duration of SRSE

Super-refractive epileptic disorder (SRSE) is one of the most serious emergencies in intensive neurology, characterized by persistent seizure activity despite the use of multi-drug antiepileptic therapy and continuous infusions of anesthetics. Long-term use of anesthetics is associated with numerous complications, such as hypotension, immunosuppression, infections, rhabdomyolysis or organ damage, and significantly prolongs the time spent in the ICU [35]. In this context, the KD is becoming an increasingly considered metabolic intervention that can support the cessation of seizure activity, allow for the gradual withdrawal of anesthetics, and shorten the duration of SRSEs. The KD induces a state of ketosis, in which the brain uses ketone bodies as the main source of energy [36]. The change in the energy substrate leads to a number of anticonvulsant effects: stabilization of neuronal membranes, modulation of ion channels, increase in GABAergic activity and reduction in glutamatergic excitation. Available studies suggest that the KD may be feasible and potentially beneficial in selected ICU populations; however, the current evidence base is limited and does not allow conclusions regarding clinical effectiveness [37]. These multidirectional effects make the KD work differently from classic antiepileptic drugs, which makes it a valuable complement to pharmacological therapy. In clinical trials, both pediatric and adult, it has been shown that most patients achieve stable ketosis within a few days of KD implementation. Importantly, a significant proportion of patients have been shown to gradually discontinue anesthetics during the first week of therapy [38].

In many case reports and cohort analyses, SRSE resolution occurred after stable ketosis was achieved, suggesting a potential causal relationship. In pediatric studies, most children were able to discontinue anesthetic infusions within 7 days of the onset of KD, while in adult studies a reduction in the duration of SRSE and a decrease in the intensity of sedation were observed. KD may also reduce the need for high doses of anesthetics, which reduces the risk of hemodynamic and metabolic complications [35,38]. This makes it possible to carry out awakening tests faster, assess the neurological condition and implement further causal treatment. Early discontinuation of anesthetics is crucial for the prognosis, as long-term deep sedation is associated with a higher risk of infections, organ failure, and prolonged mechanical ventilation [39].

Available data also indicate that earlier implementation of KD may increase the likelihood of SRSE termination and shorten its duration [37]. Although the mechanisms of this phenomenon are not fully understood, it has been suggested that the rapid achievement of ketosis may break the metabolic inflammatory vicious cycle that sustains seizure activity. The KD represents a promising intervention to support the treatment of SRSE by allowing for the gradual withdrawal of anesthetics and potentially shortening the duration of epileptic status [40]. Its unique mechanisms of action, increasing number of positive clinical observations, and acceptable safety profile indicate that KD may play an important role in the integrated treatment of SRSE. However, more research is needed to pinpoint the optimal timing of implementation, nutritional protocols, and patient groups that benefit the most [35].

### 3.5. KD May Improve Functional Neurological Outcomes

The KD has been used as an antiepileptic therapy for more than a century; however, in recent years, there has been a growing interest in its potential impact on functional neurological outcomes in critically ill patients. In the population of patients with severe neurological disorders—such as super-refractive epileptic state (SRSE), brain injuries, metabolic encephalopathies or sepsis with central nervous system involvement—profound energy, mitochondrial and inflammatory disorders are observed [41]. The KD, through its multidirectional metabolic and neuroprotective effects, can support regenerative processes and improve long-term functional outcomes.

Mechanisms of action of the KD include stabilization of neuronal membranes, modulation of ion channels, increase in GABAergic activity, and reduction in glutamatergic excitation. Ketone bodies, especially β-hydroxybutyrate, provide a more efficient fuel for neurons than glucose, generating more ATP with a lower oxidative load [42]. Under conditions of brain injury, when glycolysis is impaired and mitochondria are dysfunctional, ketones can support the maintenance of energy homeostasis and reduce secondary neuronal damage [43]. The KD also has strong anti-inflammatory and antioxidant effects. β-hydroxybutyrate inhibits the activation of the NLRP3 inflammasome, reduces the production of pro-inflammatory cytokines, and reduces oxidative stress, processes that play a key role in the progression of neurological damage. Reducing neuroinflammation may promote better neuronal regeneration, improved synaptic plasticity and better functional outcomes [44].

In clinical trials on SRSE, it has been shown that patients who achieved stable ketosis were more likely to achieve functional improvement as assessed on the mRS scale at 3 and 6 months. Although data are still limited, these observations suggest that the KD may not only interrupt seizure activity, but also affect long-term neurological recovery [41,45]. In pediatric populations, use of the KD was associated with improved cognitive function, reduced behavioral deficits, and improved return to pre-disease activity. The KD may also support the process of discontinuation of anesthetics and shorten the duration of SRSE, which indirectly affects neurological outcomes. Prolonged deep sedation is a risk factor for delirium, infections, organ failure, and prolonged mechanical ventilation—all of which worsen the neurological prognosis [35]. Early discontinuation of sedation allows for faster assessment of neurological status, implementation of rehabilitation and reduction in complications.

Preliminary data from studies on KD in sepsis indicate that ketones may improve mitochondrial function and reduce the neuroinflammatory components of septic encephalopathy, which may also translate into better cognitive and functional outcomes after leaving the ICU [46]. The KD can support the improvement of functional neurological outcomes through its neuroprotective effects, stabilization of neuronal metabolism, reduction in neuroinflammation, and indirect effect on the reduction in sedation time and duration of SRSE. While the available data are promising, more research is needed to conclusively determine in which populations and under which protocols the KD provides the greatest functional benefits [35,38,44].

### 3.6. KD May Modulate the Immune Response

The KD, traditionally used to treat drug-resistant epilepsy, has gained attention in recent years as a potential metabolic intervention affecting the functioning of the immune system. In the context of intensive care, where immune disorders—both over-inflammatory response and immunosuppression—play a key role in the pathophysiology of critical diseases, modulation of immunometabolism becomes particularly important. The KD, by inducing ketosis and changing the dominant energy substrate, may affect numerous pathways regulating the immune response [45,46].

The central element of KD action is an increase in the concentration of β-hydroxybutyrate (BHB), which has not only an energetic function, but also a signaling function. BHB inhibits the activation of the NLRP3 inflammasome—one of the main regulators of the inflammatory response—leading to a decrease in the production of pro-inflammatory cytokines such as IL-1β and IL-18. The NLRP3 inflammasome is overactivated in many critical conditions, including sepsis, trauma, and metabolic encephalopathies, therefore its modulation may reduce tissue damage and secondary immune disorders [47]. The KD also affects the function of T cells. Clinical studies have shown that patients with sepsis fed with a KD had a decrease in gene expression associated with T cell activation and signaling, which may reflect a more balanced immune response. At the same time, ketones support the survival and function of memory lymphocytes, suggesting that KD may modulate both innate and adaptive responses [48].

An important element of the action of a KD is also the effect on mitochondrial metabolism. Ketone bodies are a more efficient fuel than glucose, generating more ATP with less oxidative load. In critical illness settings, where mitochondrial dysfunction is common and leads to immunoparalysis, ketones can support the energy regeneration of immune cells [46,49]. Improving mitochondrial function promotes a more efficient immune response, reduces the production of reactive oxygen species, and reduces oxidative stress. The KD can also affect macrophage metabolism by shifting their phenotype from pro-inflammatory (M1) to more anti-inflammatory and regenerative (M2). Such modulation may be important in conditions where chronic activation of macrophages leads to tissue damage, such as in sepsis or multi-organ injuries.

In clinical trials, it has been observed that patients fed with a KD showed lower concentrations of pro-inflammatory cytokines and a more stable immune profile [50]. Although data are still limited, these results indicate that a KD may support the balance between inflammatory response and immunosuppression—a key component of the prognosis in intensive care. The KD can modulate the immune response by inhibiting the NLRP3 inflammasome, reducing pro-inflammatory cytokines, influencing T cell function, improving mitochondrial metabolism, and altering the phenotype of macrophages. These multidirectional mechanisms suggest that KD may be a valuable tool to support critical immune regulation [51]. However, more research is needed to precisely determine its role in immunomodulation and potential clinical benefits.

The evidence identified in this review is limited by small sample sizes, heterogeneous KD protocols, and the absence of randomized trials except for one study in sepsis. Consequently, the findings presented in this section should be interpreted as an evidence map rather than a basis for determining clinical efficacy.

## 4. Conclusions

This scoping review aimed to map and summarize the existing research on the use of the KD in intensive care settings. In line with the purpose of a scoping review, the objective was not to assess effectiveness of the KD but to identify the types of studies, populations, and interventions currently described in the literature. The studies analyzed showed that the KD is feasible, safe and implementable even in patients with extremely severe conditions, which is supported by the observations that “stable ketosis was achieved in all patients with KD” and that “no major adverse events or harmful metabolic adverse reactions were observed”. The strongest evidence concerns the use of a KD in the treatment of refractive and super-refractive epileptic states (RSE/SRSE), where this diet may support the suppression of seizure activity, enable reduction in anesthetic doses, and shorten the duration of SRSE. In many studies, patients achieved stable ketosis within a few days, which often correlated with improved seizure control and the ability to discontinue anesthetic infusions.

KD may also support metabolic control in patients with sepsis by reducing insulin demand, stabilizing glycemia, and modulating the immune response. One study showed that “after day 4, none of the patients in the KD group required insulin treatment,” highlighting the potential of a KD as an intervention to improve metabolic homeostasis in the course of severe disease. Despite the promising results, the quality of the available evidence remains limited, with most studies being retrospective, involving small groups of patients and with significant heterogeneity.

There is also a lack of standardized protocols for the implementation of KD in intensive care settings, which makes it difficult to compare outcomes and formulate unambiguous clinical recommendations. In conclusion, the KD appears to be a valuable, non-pharmacological metabolic strategy that can support the treatment of neurological disorders, stabilize glucose metabolism, and modulate the inflammatory response in ICU patients. Although the available evidence indicates that the KD may be feasible and generally well tolerated in ICU patients, further well-designed prospective and randomized studies are required to determine its potential clinical effectiveness and to identify the patient groups most likely to benefit.

In particular, the lack of randomized controlled trials—apart from a single RCT conducted in septic patients—significantly limits the ability to draw firm conclusions regarding the efficacy of the KD across different ICU populations. Future research should prioritize randomized designs to establish standardized protocols and identify patient groups that may benefit most.

## 5. Limitations

This scoping review has several important limitations that need to be taken into account when interpreting the results. First, the number of studies available on the use of the KD (CD) in intensive care patients is small, and most of them involve small groups of patients, which limits the possibility of generalizing the results. Many publications have only looked at single centers or specific clinical populations, such as patients with super-refractive epilepsy or sepsis, which further narrows the scope of inference. A significant proportion of the included studies are retrospective or descriptive, and only a few papers include prospective KD implementation protocols.

The lack of randomized clinical trials makes it difficult to assess the actual efficacy and safety of the KD in intensive care. In addition, the heterogeneity of research projects, differences in nutritional protocols and different definitions of endpoints limit the ability to compare results between studies. Many publications do not provide detailed data on metabolic monitoring, adverse reactions or discontinuation criteria. While one study highlighted that “no major adverse events or harmful metabolic adverse events were observed,” the lack of standardization of reporting makes it difficult to assess the full safety profile of the KD in ICU. Differences in how ketosis is defined and monitored can also affect the interpretation of results.

The review only included publications available in English, which may lead to the omission of studies published in other languages. In addition, some of the papers were excluded due to lack of access to the full text, which may have limited the scope of the analysis. The selection process, although carried out in accordance with JBI and PRISMAScR guidelines, was based on available summaries and full texts, which is always associated with the risk of unintentional omission of relevant data. Overall, while the results of the review point to potential benefits of using the KD in intensive care, the limitations of the available data underscore the need for further, well-designed prospective and randomized trials.

Despite the growing clinical interest in ketogenic nutrition in the ICU, the strength of the current evidence remains constrained by the scarcity of randomized controlled trials. Among the studies included in this review, only one RCT—conducted in patients with sepsis—met the criteria for high-level evidence. All remaining publications were observational or based on clinical experience. This imbalance reflects the early stage of research in this field and highlights the need for well-designed prospective trials to validate the promising findings reported so far.

## Figures and Tables

**Table 1:** Summary search of the review.

Database	Date of Search	Search String (Keywords + Boolean Operators)	Controlled Vocabulary (MeSH/Subject Headings)	Filters Applied
PubMed	10–19 April 2026	“KD” OR “KD” OR “ketogenic nutrition” AND “intensive care” OR “ICU” OR “critical illness” OR “critical care” AND “sepsis” OR “SRSE” OR “super-refractory status epilepticus” OR “refractory status epilepticus” AND “benefits” OR “metabolic effects” OR “glycemic control”	KD[MeSH], Intensive Care Units[MeSH], Critical Illness[MeSH], Status Epilepticus[MeSH], Sepsis[MeSH]	Humans; English; Full text
Scopus	10–19 April 2026	TITLE-ABS-KEY “KD” AND “intensive care” OR ICU OR “critical illness” AND (sepsis OR SRSE OR RSE AND benefits OR outcomes OR metabolism	Scopus Subject Headings (KD; Intensive care; Critical illness; Status epilepticus; Sepsis)	English; Article; Reviews; Full text
Web of Science	10–19 April 2026	TS = “KD” AND “intensive care” OR ICU OR “critical illness” AND sepsis OR SRSE OR RSE) AND (benefits OR outcomes OR metabolism	WoS Categories: Clinical Neurology; Critical Care Medicine; Nutrition & Dietetics	English; Article; Review
EBSCOhost (CINAHL/MEDLINE)	10–19 April 2026	“KD” OR “ketogenic therapy” AND “intensive care” OR ICU OR “critical illness” AND sepsis OR SRSE OR RSE	CINAHL Headings: KD; Intensive Care Units; Critical Illness; Status Epilepticus; Sepsis	Humans; English; Peer-reviewed
Cochrane Library	10–19 April 2026	“KD” AND “intensive care” OR “KD” AND SRSE OR “KD” AND sepsis	Cochrane Index Terms: KD; Intensive Care; Status Epilepticus; Sepsis	Trials; Reviews; English
Google Scholar	10–19 April 2026	“KD in ICU” OR “KD critical illness” OR “KD sepsis” OR “KD SRSE”	Not applicable	No filters (manual screening of first 200 results

**Table 2:** Characteristics and findings of studies included in this review.

Author, Year	Country	Aim of the Study	Participants	Results and Findings	KD Formula/Ratio/Mode of Administration	Adverse Effects (Specific)
Rahmela T. et al., 2024 [1]	Germany	to verify that patients with sepsis in intensive care can safely and effectively induce stable ketosis through a KD	40 critically ill patients with sepsis	✓ stable ketosis was achieved in all patients with a KD ✓ no major adverse events or harmful metabolic adverse reactions (acidosis, dysglycemia or dyslipidemia) ✓ after day 4, none of the patients in the KD group required insulin treatment there were no differences in 30-day survival in KD patients vs. control ✓ analysis of T-lymphocytes and cytokines showed less immune deregulation, reduced expression of genes associated with T cell activation and signaling, lower secretion of pro-inflammatory cytokines	- enteral KD, a classic high-fat formula (details in the article)	mild acidosis, dysglycemia, dyslipidemia; no major adverse events
Park EG. et al., 2019 [5]	Korea	evaluation of experience with a KD for patients with SRSE in intensive care units	retrospectively reviewed the medical records of 16 patients (10 males, 6 females) with SRSE who were treated with the KD in the ICUs	✓ a KD is an effective alternative therapeutic strategy for patients with SRSE in ICUs with adequate efficacy and safety in reducing seizure frequency and weaning from prolonged mechanical ventilation	- the classic KD used in SRSE; usually 3:1 or 4:1 (according to the ICU protocol)	hypoglycemia, hyperlipidemia, metabolic acidosis, GI intolerance, constipation
Feil K. et al., 2025 [6]	Germany	creating a standard management procedure (SOP) for the use of the classic KD (CD) in adult patients in the intensive care unit with refractive epileptic state (RFE) and super-refractive epileptic state (SRSE)	-	✓ early initiation of the KD is recommended in all patients with SRSE without contraindications, taking into account its safety, reversibility and rapid implementation	- classic KD 4:1 according to SOP	hypoglycemia, hyperlipidemia, metabolic acidosis, electrolyte disturbances, GI intolerance
Ren Y. et al., 2025 [4]	China	to assess the feasibility, safety and efficacy of a KD for patients with super-refractive epileptic state (SRSE) in the intensive care unit (ICU)	twelve patients (9 females and 3 males) with SRSE	✓ a KD may be effective in treating SRSE in the ICU, and it is also relatively safe ✓ however, there are many adverse events that can be corrected with close monitoring	- classic KD 4:1, enteral feeding	metabolic acidosis, hyperlipidemia, hypoglycemia, GI intolerance, electrolyte abnormalities
Worden LT. et al., 2020 [2]	USA	retrospective cohort study of subsequent patients with CD started in the ICU from 2010 to 2018 for SRSE and EE	29 pediatric patients from ICU	✓ ICU CD is feasible, safe and often effective for SRSE and EE ✓ adverse reactions were common but treatable ✓ a reduction in seizure dose of ≥50% in most patients with a response to treatment is achieved within 1–2 weeks	- pediatric classic KD, usually 3:1 or 4:1; enteral or NG tube	hypoglycemia, hyperlipidemia, metabolic acidosis, electrolyte disturbances, GI intolerance
Farias-Moeller R. et al., 2017 [12]	USA	to describe and evaluate the feasibility of a practical way to implement a KD in children with super-refractive epileptic state (SRSE) in a pediatric intensive care unit (PICU)	9 children with super-refractive epileptic state (SRSE), patients hospitalized in a pediatric intensive care unit	✓ implementing a KD in children with SRSE in PICU is feasible ✓ most children may have been weaned off continuous anesthetic infusions within a week, suggesting that a KD may support seizure control in SRSE ✓ adverse reactions occurred, but manageable with appropriate supervision.	- classic KD, enteral, prepared in hospital	vomiting, metabolic acidosis, hypoglycemia, hyperlipidemia, feeding intolerance
Feil K. et al., 2026 [13]	Germany	to evaluate the feasibility, safety and clinical effect of the KD in adult patients with super-refractive epileptic state (SRSE)	34 adult patients with SRSE (18 patients treated with a KD, 16 patients in the control group	✓ a KD is feasible and safe in adult patients with SRSE ✓ results support a prospective assessment of the KD as a non-pharmacological therapy in neurocritical care	- classic KD 4:1 according to SOP	metabolic acidosis, hyperlipidemia, hypoglycemia, GI intolerance, electrolyte disturbances

**Table 3:** Comparison of clinical domains: SRSE vs. sepsis vs. glycemic control vs. general metabolic effects of KD in ICU.

Clinical Area	Key Results KD	Mechanisms of Action	Safety and Tolerance
SRSE (Super-refractory status epilepticus) [2,3,4,5,12,13]	• Reduction in seizure activity • Possibility of discontinuation of anesthetics • Reduction in SRSE duration • Efficacy in refractory cases	• Stabilization of neuronal membranes • Modulation of ion channels • ↑ GABA, ↓ glutamate • Neuroprotection and ↓ oxidative stress • Improvement of mitochondrial functions	Safety profile acceptable; mild and manageable side effects
Sepsis [1]	• Glycemic stabilization • Reduction in insulin demand • Improvement of immunometabolism • Reduction in immune deregulation	• Inhibition of NLRP3 • ↓ pro-inflammatory cytokines • Improvement of oxidative phosphorylation • Ketones as an efficient metabolic fuel	No serious side effects; good metabolic tolerance
Glycemic control in the ICU [1]	• Reduction in glycemic fluctuations • Significant reduction in insulin requirements • Possibility of complete insulin withdrawal • More stable metabolic profile	• Limitation of glycolysis • ↑ insulin sensitivity • ↓ visceral glucose production • Modulation of the inflammatory response	Safety profile comparable to standard feeding; absence of ketoacidosis
General metabolic effects of KD in ICU [1,6]	• Improving cellular bioenergetics • Increasing the efficiency of ATP production • Reducing oxidative stress • Modulating the inflammatory response • Stabilizing metabolism in organ failure	• Ketones as an alternative fuel with higher energy efficiency • ↓ production of reactive oxygen species • Improvement of mitochondrial function • Regulation of immunometabolic pathways	KD feasible even in patients in extremely severe condition; controllable side effects

*↑—increase; ↓—decline.*

## Data Availability

Not applicable.

## Supplementary Materials

The following supporting information can be downloaded at: https://www.mdpi.com/article/10.3390/nu18121943/s1, Table S1 Preferred Reporting Items for Systematic reviews and Meta-Analyses extension for Scoping Reviews (PRISMA-ScR) Checklist.

## Abbreviations

The following abbreviations are used in this manuscript:

ICU	intensive care units
KD	KD
SRSE	super-refractive epileptic state
PCC	Population–Concept–Context
RSE	Refractory status epilepticus
EE	Energy Expenditure
NLRP3	NOD-like receptor pyrin domain-containing protein 3
BHB	β-hydroxybutyrate

## References

[1] Borowicz-Reutt K., Krawczyk M., Czernia J. (2024). Ketogenic Diet in the Treatment of Epilepsy. Nutrients.

[2] Martin-McGill K.J., Bresnahan R., Levy R.G., Cooper P.N. (2020). Ketogenic diets for drug-resistant epilepsy. Cochrane Database Syst. Rev..

[3] McNally M.A., Hartman A.L. (2012). Ketone bodies in epilepsy. J. Neurochem..

[4] Ren Y., Zhang M., Fu X., Zhang Y., Liu F., Wu C., Shi H., Tian F., Liu G., Lin Y. (2025). Ketogenic diet treatment for super-refractory status epilepticus in the intensive care unit: Feasibility, safety and effectiveness. Front. Neurol..

[5] Park E.G., Lee J., Lee J. (2019). The ketogenic diet for super-refractory status epilepticus patients in intensive care units. Brain Dev..

[6] Feil K., Schweikert D., Adolph M., Kindzierski S., Single C., Becker F., Bösel J., Möller L., Mengel A. (2025). Ketogenic diet for status epilepticus in adult intensive care unit patients: A standard operating procedure. Neurol. Res. Pract..

[7] Wang Z., Chen T., Wu S., Dong X., Zhang M., Ma G. (2024). Impact of the ketogenic diet as a dietary approach on cardiovascular disease risk factors: A meta-analysis of randomized clinical trials. Am. J. Clin. Nutr..

[8] Munn Z., Peters M., Stern C., Tufanaru C., McArthur A., Aromataris E. (2018). Systematic review or scoping review? Guidance for authors when choosing between a systematic or scoping review approach. BMC Med. Res. Methodol..

[9] Peters M., Godfrey C., McInerney P., Baldini Soares C., Khalil H., Parker D. (2015). The Joanna Briggs Institute Reviewers’ Manual 2015: Methodology for JBI Scoping Reviews.

[10] Tricco A.C., Lillie E., Zarin W., O’Brien K.K., Colquhoun H., Levac D., Moher D., Peters M.D.J., Horsley T., Weeks L. (2018). PRISMA extension for scoping reviews (PRISMA-ScR): Checklist and explanation. Ann. Intern. Med..

[11] Peters M.D.J., Godfrey C., McInerney P., Munn Z., Tricco A., Khalil H., Aromataris E., Munn Z. (2020). Chapter 11: Scoping Reviews (2020 version). JBI Reviewer’s Manual.

[12] Farias-Moeller R., Bartolini L., Pasupuleti A., Brittany Cines R.D., Kao A., Carpenter J.L. (2017). A Practical Approach to Ketogenic Diet in the Pediatric Intensive Care Unit for Super-Refractory Status Epilepticus. Neurocrit. Care.

[13] Feil K., Kindzierski S., Single C., Geiger-Primo L., Schweikert D., Adolph M., Kegele J., Lerche H., Ziemann U., Möller L. (2026). Ketogenic Diet in Super-Refractory Status Epilepticus: A Retrospective Cohort Study with Severity-Matched Controls in Critically Ill Adults. Neurocrit. Care.

[14] Kaul N., Laing J., Nicolo J.P., Nation J., Kwan P., O’Brien T.J. (2021). Practical Considerations for Ketogenic Diet in Adults with Super-Refractory Status Epilepticus. Neurol. Clin. Pract..

[15] Nabbout R., Matricardi S., De Liso P., Dulac O., Oualha M. (2023). Ketogenic diet for super-refractory status epilepticus (SRSE) with NORSE and FIRES: Single tertiary center experience and literature data. Front. Neurol..

[16] Cornwall C.D., Krøigård T., Kristensen J.S.S., Callesen H.E., Beier C.P. (2023). Outcomes and Treatment Approaches for Super-Refractory Status Epilepticus: A Systematic Review and Meta-Analysis. JAMA Neurol..

[17] Thompson L., Fecske E., Salim M., Hall A. (2017). Use of the ketogenic diet in the neonatal intensive care unit-Safety and tolerability. Epilepsia.

[18] Camões J., Reis A.H., Sousa L., Gomes E. (2021). Super-refractory status epilepticus and ketogenic diet in intensive care: A series report. Rev. Bras. Ter. Intensiv..

[19] Sukkar S.G., Cogorno L., Pisciotta L., Pasta A., Vena A., Gradaschi R., Dentone C., Guiddo E., Martino E., Beltramini S. (2021). Clinical efficacy of eucaloric ketogenic nutrition in the COVID-19 cytokine storm: A retrospective analysis of mortality and intensive care unit admission. Nutrition.

[20] D’Amato G., Gentile M., Carella R., Giannini A., Faienza M.F., Tummolo A. (2026). The Ketogenic Diet in the Neonatal Intensive Care Setting: The Case of a Preterm Newborn with Mitochondrial DNA Depletion Syndrome Type 13 (MTDPS13). Case Rep. Genet..

[21] Breu M., Häfele C., Glatter S., Trimmel-Schwahofer P., Golej J., Male C., Feucht M., Dressler A. (2021). Ketogenic Diet in the Treatment of Super-Refractory Status Epilepticus at a Pediatric Intensive Care Unit: A Single-Center Experience. Front. Neurol..

[22] Francis B.A., Fillenworth J., Gorelick P., Karanec K., Tanner A. (2019). The Feasibility, Safety and Effectiveness of a Ketogenic Diet for Refractory Status Epilepticus in Adults in the Intensive Care Unit. Neurocrit. Care.

[23] Miao Y., Xie L., Chen S., Zhang X., Liu W., Xie P. (2024). Ketogenic diet in treating sepsis-related acquired weakness: Is it friend or foe?. Front. Nutr..

[24] Wang M. (2024). Ketogenic diet benefits in critically ill patients with sepsis. Nat. Rev. Nephrol..

[25] Falsaperla R., Sortino V., Collotta A.D., Privitera G.F., Palmeri A., Mauceri L., Ruggieri M. (2023). Ketogenic Diet in Neonates with Drug-Resistant Epilepsy: Efficacy and Side Effects-A Single Center’s Initial Experience. Neuropediatrics.

[26] Muscogiuri G., El Ghoch M., Colao A., Hassapidou M., Yumuk V., Busetto L., Obesity Management Task Force (OMTF) of the European Association for the Study of Obesity (EASO) (2021). European Guidelines for Obesity Management in Adults with a Very Low-Calorie Ketogenic Diet: A Systematic Review and Meta-Analysis. Obes. Facts..

[27] Buga A., Kackley M.L., Crabtree C.D., Bedell T.N., Robinson B.T., Stoner J.T., Decker D.D., Hyde P.N., LaFountain R.A., Brownlow M.L. (2023). Fasting and diurnal blood ketonemia and glycemia responses to a six-week, energy-controlled ketogenic diet, supplemented with racemic R/S-BHB salts. Clin. Nutr. ESPEN.

[28] Lodi A., Zarantonello L., Bisiacchi P.S., Cenci L., Paoli A. (2020). Ketonemia and Glycemia Affect Appetite Levels and Executive Functions in Overweight Females During Two Ketogenic Diets. Obesity.

[29] Thakur K.T., Probasco J.C., Hocker S.E., Roehl K., Henry B., Kossoff E.H., Kaplan P.W., Geocadin R.G., Hartman A.L., Venkatesan A. (2014). Ketogenic diet for adults in super-refractory status epilepticus. Neurology.

[30] Katz J.B., Owusu K., Nussbaum I., Beekman R., DeFilippo N.A., Gilmore E.J., Hirsch L.J., Cervenka M.C., Maciel C.B. (2021). Pearls and Pitfalls of Introducing Ketogenic Diet in Adult Status Epilepticus: A Practical Guide for the Intensivist. J. Clin. Med..

[31] Cervenka M.C., Hartman A.L., Venkatesan A., Geocadin R.G., Kossoff E.H. (2011). The Ketogenic Diet for medically and surgically refractory status epilepticus in the neurocritical care unit. Neurocrit. Care.

[32] Cobo N.H., Sankar R., Murata K.K., Sewak S.L., Kezele M.A., Matsumoto J.H. (2015). The Ketogenic Diet as broad-spectrum treatment for super-refractory pediatric status epilepticus: Challenges in implementation in the pediatric and neonatal intensive care units. J. Child Neurol..

[33] Borhan M.K., Vethakkan S.R., Sarvanandan T., Paramasivam S.S. (2023). A Case of Severe Lactation Ketoacidosis in a Nondiabetic Mother on a Ketogenic Diet. JCEM Case Rep..

[34] Pizzo F., Collotta A.D., Di Nora A., Costanza G., Ruggieri M., Falsaperla R. (2022). Ketogenic diet in pediatric seizures: A randomized controlled trial review and meta-analysis. Expert Rev. Neurother..

[35] Cenci L., Paoli A., Omar H.R., Dalvi P., Camporesi E.M., Mangar D., Quartesan S., Fiorito A., Bosco G. (2018). Internist, anesthesiologist and surgeon use of ketogenic diet. Minerva Gastroenterol. Dietol..

[36] Alolayan Y.S., McKinley K., Bhatia R., Alkhachroum A. (2021). Review and Updates on the Treatment of Refractory and Super Refractory Status Epilepticus. J. Clin. Med..

[37] Smith G., Press C.A. (2017). Ketogenic Diet in Super-Refractory Status Epilepticus. Pediatr. Neurol. Briefs.

[38] Allen C.M., Hall C.A., Cox N.E., Ryan H., De Beer T., O’Donoghue M.F. (2021). Adjunctive use of the ketogenic diet in a young adult with UBE2A deficiency syndrome and super-refractory status epilepticus. Epilepsy Behav. Rep..

[39] Arora N., Litofsky N.S., Golzy M., Aneja R., Staudenmyer D., Qualls K., Patil S. (2022). Phase I single center trial of ketogenic diet for adults with traumatic brain injury. Clin. Nutr. ESPEN.

[40] Bassuel C., Michot M., Dabernat S., Bats M.L. (2026). A toothache gone wrong: An unexpected case of perioperative lactation ketoacidosis. Int. Breastfeed. J..

[41] Ułamek-Kozioł M., Czuczwar S.J., Januszewski S., Pluta R. (2019). Ketogenic Diet and Epilepsy. Nutrients.

[42] Haridas B., Testino A., Kossoff E.H. (2025). Ketogenic diet therapy for the treatment of pediatric epilepsy. Epileptic Disord..

[43] Gras M., Bearden D., West J., Nabbout R. (2024). Efficacy of anti-seizure medications and alternative therapies (ketogenic diet, CBD, and quinidine) in KCNT1-related epilepsy: A systematic review. Epilepsia Open.

[44] Chomtho S., Uaariyapanichkul J., Chomtho K. (2022). Outcomes of parenteral vs. enteral ketogenic diet in pediatric super-refractory status epilepticus. Seizure.

[45] Prasoppokakorn T., Jirasakuldej S., Lakananurak N. (2019). Medium-chain triglyceride ketogenic diet is effective for treatment of an adult with super-refractory status epilepticus: A case report and literature review. Eur. J. Clin. Nutr..

[46] Arya R., Peariso K., Gaínza-Lein M., Harvey J., Bergin A., Brenton J.N., Burrows B.T., Glauser T., Goodkin H.P., Lai Y.C. (2018). Efficacy and safety of ketogenic diet for treatment of pediatric convulsive refractory status epilepticus. Epilepsy Res..

[47] Duan R., Wang T., Li Z., Jiang L., Yu X., He D., Tao T., Liu X., Huang Z., Feng L. (2024). Ketogenic diet modulates immune cell transcriptional landscape and ameliorates experimental autoimmune uveitis in mice. J. Neuroinflamm..

[48] Monda A., La Torre M.E., Messina A., Di Maio G., Monda V., Moscatelli F., De Stefano M., La Marra M., Padova M.D., Dipace A. (2024). Exploring the ketogenic diet’s potential in reducing neuroinflammation and modulating immune responses. Front. Immunol..

[49] Link V.M., Subramanian P., Cheung F., Han K.L., Stacy A., Chi L., Sellers B.A., Koroleva G., Courville A.B., Mistry S. (2024). Differential peripheral immune signatures elicited by vegan versus ketogenic diets in humans. Nat. Med..

[50] Peng H., Fu M., Li J. (2025). New perspectives: The impact of ketogenic diet on the immune system. Signal Transduct. Target. Ther..

[51] Shaw D.M., Merien F., Braakhuis A., Keaney L., Dulson D.K. (2021). Adaptation to a ketogenic diet modulates adaptive and mucosal immune markers in trained male endurance athletes. Scand. J. Med. Sci. Sports.

